# Phage idiotype vaccination: first phase I/II clinical trial in patients with multiple myeloma

**DOI:** 10.1186/1479-5876-12-119

**Published:** 2014-05-09

**Authors:** Tim Roehnisch, Cornelia Then, Wolfgang Nagel, Christina Blumenthal, Todd Braciak, Mariel Donzeau, Thomas Böhm, Michael Flaig, Carole Bourquin, Fuat S Oduncu

**Affiliations:** 1 Division of Hematology and Oncology, Medizinische Klinik und Poliklinik IV, Klinikum der Universität München, Munich, Germany; 2 Division of Endocrinology and Diabetology, Medizinische Klinik und Poliklinik IV, Klinikum der Universität München, Munich, Germany; 3 Helmholtz Zentrum München, Deutsches Forschungszentrum für Gesundheit und Umwelt, Munich, Germany; 4 Maître de conférences (MCF) - Université de Strasbourg, Unité UMR Biotechnologie et Signalisation Cellulaire, Strasbourg, France; 5 Dermatologische Klinik der Universität München, Munich, Germany; 6 Université de Fribourg, Département de Médecine, Chair of Pharmacology, Fribourg, Switzerland

**Keywords:** Patient-specific immunotherapy, Phage idiotype vaccination, Multiple myeloma, Phase I/II clinical trial, Paraprotein

## Abstract

**Background:**

Multiple myeloma is characterized by clonal expansion of B cells producing monoclonal immunoglobulins or fragments thereof, which can be detected in the serum and/or urine and are ideal target antigens for patient-specific immunotherapies.

**Methods:**

Using phage particles as immunological carriers, we employed a novel chemically linked idiotype vaccine in a clinical phase I/II trial including 15 patients with advanced multiple myeloma. Vaccines composed of purified paraproteins linked to phage were manufactured successfully for each patient. Patients received six intradermal immunizations with phage idiotype vaccines in three different dose groups.

**Results:**

Phage idiotype was well tolerated by all study participants. A subset of patients (80% in the middle dose group) displayed a clinical response indicated by decrease or stabilization of paraprotein levels. Patients exhibiting a clinical response to phage vaccines also raised idiotype-specific immunoglobulins. Induction of a cellular immune response was demonstrated by a cytotoxicity assay and delayed type hypersensitivity tests.

**Conclusion:**

We present a simple, time- and cost-efficient phage idiotype vaccination strategy, which represents a safe and feasible patient-specific therapy for patients with advanced multiple myeloma and produced promising anti-tumor activity in a subset of patients.

## Background

Due to the prospect of effective tumor therapies with minimal adverse events, anti-tumor vaccines have long been investigated. A successful example is the passive vaccination against B cell lymphoma with the anti-CD20 antibody rituximab [1]. However, rituximab targets CD20 expressed not only on lymphoma cells but normal B cells as well. A personalized active vaccination strategy targeting a tumor-specific antigen may accomplish an even better and more sustained therapeutic response.

An easily identifiable tumor-specific antigen, from which a patient-specific vaccine could be generated, is the variable region of the clonal immunoglobulin (idiotype, Id) expressed on the surface of B cell cancers, being unique to each neoplastic B cell clone. However, provoking sufficient immunogenicity of the Id, which represents a tumor-specific antigen [2], but nevertheless is a self-protein, is a still unmet challenge. Previously, Id was usually coupled to a strong immunogenic carrier protein, such as keyhole limpet hemocyanin (KLH), and co-administered with immunostimulatory adjuvants, such as granulocyte-monocyte colony stimulating factor (GM-CSF) [3,4]. Despite encouraging preclinical results, Id-based immunotherapy so far has given disappointing outcomes in patients; i.e. clinical phase III studies aimed at obtaining regulatory approval for Id-KLH vaccines failed to reach their primary endpoints [5,6]. Utilizing the immunogenic properties of the filamentous phage, we previously demonstrated a superior immunogenicity of a chemically linked Id-phage compared to Id-KLH and a genetically engineered Id-phage in the preclinical setting [7]. We here examine the therapeutic feasibility and tolerability of the chemically linked Id-phage in patients with advanced multiple myeloma (MM).

## Methods

### Multiple myeloma Id purification

Paraproteins were purified from serum and urine samples according to their heavy-chain isotype and subclass employing protein A affinity chromatography followed by ion exchange chromatography on an ÄKTA Purifier 10 using Unicorn 4.11 software (Amersham Biosciences, Braunschweig, Germany) as described previously [7]. Briefly, serum (500 μl) was passed through 0.8 μm and 0.2 μm nitrocellulose filters equilibrated with 500 μl 20 mM tetra-sodium diphosphate buffer (pH 6.4; Merck, Darmstadt, Germany) and bound to a HiTrap Protein A HP/5 ml column (Amersham Biosciences). Individual IgG fractions were eluted using a pH step gradient (100 mM sodium citrate/150 mM NaCl/pH 3.5; Merck), dialyzed against 20 mM Tris/HCl/pH 8.5 and bound to HiTrap Q-HP/5 ml column (Amersham Biosciences) equilibrated with 20 mM Tris/HCl/pH 8.5 (Merck, Darmstadt, Germany). Samples were eluted with 20 mM Tris/1 M NaCl/pH 8.5 (Merck) using a linear salt gradient and the paraprotein was dialyzed against phosphate-buffered saline (PBS; Invitrogen, Karlsruhe, Germany). The purified paraprotein was sterile filtered through a 0.2 μm nitrocellulose.

### Preparation of Id vaccines

The preparation of bacteriophages (M13K07, Amersham Biosciences) at large scale was performed as described previously [7]. Contaminating endotoxins were removed by two-phase Triton X-114 separation as described elsewhere [8,9], resulting in an endotoxin concentration of < 1 unit/ml [7]. Chemically linked Id-phage was generated as described [7] by coupling purified paraproteins to bacteriophages (50 mg/ml in PBS) with 0.1% (v/v) glutaraldehyde/water and an Id protein/phage ratio of 1:10 (w/w). Phage vaccines were sterile filtered and filled according to the work-flow by the pharmacy of the Klinikum Innenstadt (Ludwig-Maximilians-Universität, Munich). Sterility tests were performed according to standard procedures by the Max von Pettenkofer-Institut (Ludwig-Maximilians-Universität, Munich).

### Safety and toxicity

Preclinical toxicity studies were performed in cooperation with an independent partner (Bioservice Scientific Laboratories GmbH, Planegg, Germany) in compliance with the principles of Good Laboratory Practice. A wildtype phage dosage of 10 ml/kg (rat, n = 6) and 50 ml/kg body weight (mouse, n = 6) intraperitoneally and subcutaneously resulted in no clinical signs of toxicity throughout the observation period of 14 days. In New Zealand white rabbits, intradermal and intramuscular application (0.2 ml) provoked a slight acute reactionary inflammatory heterophil cell response with moderate hemorrhage detected in all rabbits (n = 14), lacking necrosis or ulceration. Overall, histopathological responses at the injection site were slight, suggesting that significant toxicity would not occur in humans at this dosage.

### Enzyme-linked immunosorbent assay (ELISA)

ELISA analyses were performed as described [7]. Detection antibodies (goat-anti-human IgG/IgG1/IgG2/IgG3/IgG4) were purchased from Southern Biotech, Birmingham, USA.

### Study design and participants

The clinical study was designed as a phase I/II trial primarily addressing the feasibility and safety of phage Id vaccinations in MM patients. The local Institutional Review Board of the University of Munich approved the study. This study was announced to the Paul-Ehrlich-Institut (Langen, Germany) according to paragraph 40, section 1, no. 6 AMG and the Government of Upper Bavaria (Regierung Oberbayern, Germany) according to paragraph 67, section 1, no. 6 AMG (Study-No.: MKI-TR02/2002). All participants gave written informed consent. Seventeen patients > 18 years of age suffering from a terminal stage of MM (as defined by partial response or relapsed disease more than three months after high dose melphalan chemotherapy (at a dose between 140 and 200 mg/m^2^) and autologous stem cell transplantation) were recruited within one year. Patients had all undergone one or two high dose chemotherapies followed by autologous stem cell transplantation. Further treatment included lenalidomide, bendamustine, cyclophosphamide, thalidomide or bortezomib. All patients had been treated with corticosteroids. Patients received no chemotherapy, steroids or other myeloma-related drugs three months prior to study enrolment. Criteria for relapse were a M gradient < 2 g/dl that progressed less than 0.2 g/dl per month or an increase of urine light chain excretion > 100 mg/24 hours within one month. Further inclusion criteria were at least one positive Multitest-Immigon test (Biosyn, Fellbach, Germany), general condition according to *Eastern Cooperative Oncology Group* performance status score < 2 (Karnowsky index ≥ 70%) and written informed consent.

Exclusion criteria were active infections or autoimmune diseases, intake of thalidomide, lenalidomide, interferon alpha, dexamethasone, hydrocortisone or other immunosuppressive or non-approved drugs within 6 weeks prior to study inclusion; MM stage II or III according to Salmon & Durie requiring therapy, extramedullar myeloma, leukocyte count < 2,500/μl, CD45^+^ cell count < 1200/μl, CD3^+^ cells < 700/μl, CD4^+^ cells < 500/μl, CD8^+^ cells < 200/μl, CD4^+^/CD8^+^ ratio < 1, hemoglobin < 8 g/dl, heart failure, therapy-refractory hypertension, therapy-refractory diabetes mellitus, chronic lung disease with a FeV1 < 50% or diffusion capacity < 50%, bilirubin > 2.0 mg/dl, liver transaminases > three times the upper limit, glomerular filtration rate < 30 ml/min, previous organ transplantation other than autologous stem cell transplantation, participation in another study, severe mental disease or insufficient willingness to cooperate. Two patients were excluded due to an infringement of the eligibility criteria caused by active hepatitis and tumor-related anemia.

### Patient vaccination

Patients received a total of six intradermal immunizations with the phage-conjugated Id protein vaccine at day 1, 7, 14 and week 4, 8 and 12. The vaccine dose was 0.25 mg for patients 1–5 and was subsequently escalated to 1.25 mg for patients 6–10 and 2.5 mg for patients 11–15 as no serious adverse events were observed (equal to 1 × 10^11^ – 2.5 × 10^12^ bacteriophages). Vaccine doses of 1 × 10^10^, 1 × 10^11^ and 5 × 10^11^ were previously tested successfully in the murine BCL1 lymphoma model, with the best anti-phage antibody response after application of 5 × 10^11^ bacteriophages [7]. Each vaccine formulation additionally contained 100 μg/m^2^ GM-CSF (Leukomax®, Novartis) as adjuvant and 0.2 mg KLH (Immucothel®, Biosyn Corporation, Carlsbad, CA, USA) as control antigen. As cytokine adjuvant, 100 μg/m^2^ GM-CSF was administered close to the vaccine injection site subcutaneously for three consecutive days after the vaccination. Fifteen patients were treated with at least three vaccinations.

### Determination of cytotoxicity

Cytotoxic T lymphocyte (CTL) activity was determined employing the single cell-based fluorogenic cytotoxicity assay (CyToxiLux assay, OncoImmunin Inc, Gaithersburg, USA). Freshly thawed peripheral blood mononuclear cells (PBMCs), isolated by Ficoll-gradient centrifugation of heparinized blood from MM patients obtained at different times of vaccination, were used as effector cells. Bone marrow cells obtained from the patient before vaccine treatment were used as target cells. 1 ml of frozen bone marrow target cells were rapidly thawed at 37°C, transferred to a reagent tube containing 9 ml of complete RPMI, 10% (v/v) fetal calf serum, spun for 8 minutes at 1500 rpm, re-suspended in medium at 2 × 10^6^/ml and incubated with target cell marker at 1 μl/ml at 37°C for 1 hour, washed twice with a 10-fold volume of medium and adjusted to 2 × 10^6^ cells/ml. The effector PBMCs were thawed and re-suspended (1 × 10^8^/ml). Target cell suspension (100 μl) was dispensed with effector cells in effector-target ratios of 50:1, 25:1, 12:1 and 5:1. Controls were PBMCs and bone marrow cells alone adjusted with medium to a final volume of 200 μl. After incubation for 2 hours at 37°C and 5% CO_2_, samples were subjected to caspase substrate reaction for 30 minutes at 37°C. After washing, the samples were analyzed by flow cytometry.

### Delayed type hypersensitivity test

Phage Id vaccine, KLH, wild type phage and tumor Id were injected intracutaneously at a dose of 20 μg each four weeks after the last vaccination. The delayed type hypersensitivity reaction was determined 48 hours after the challenge by monitoring reddening, swelling and induration. An induration > 5 mm in diameter was considered minor positive (“+”). An induration > 10 mm in diameter was considered positive (“++”), an induration > 15 mm in diameter was considered strongly positive (“+++”).

### Skin biopsy

Patient 7 was chosen to undergo a skin biopsy of the site of phage Id challenge, since this patient displayed a positive Id-related delayed type hypersensitivity reaction and an Id-specific humoral response. Staining was performed with hematoxylin-eosin and anti-CD8 (Dako GmbH, Hamburg, Germany) and anti-CD4 (Novocastra, Berlin, Germany) antibodies for immunohistochemistry analysis.

## Results

### Patient characteristics, toxicity and feasibility of phage Id vaccine

The characteristics of the study participants are shown in Table 1. All 15 vaccines were manufactured successfully within a mean production time of two weeks for 0.25 and 1.25 mg and three weeks for 2.5 mg vaccine dosages. Phage vaccination was well tolerated with only minor and transient adverse events, such as skin irritation at the vaccine injection site and flu-like symptoms. All observed adverse events are summarized in Additional file 1. There was no dose-limiting toxicity at the three dose levels tested.

**Table 1:** Baseline characteristics of the study participants

**No.**	**Gender**	**Age**	**Myeloma isotype**	**Vaccination dose level**
1	M	62	λ-light chain	0.25 mg
2	M	58	IgA_1_-κ/light chain	0.25 mg
3	M	56	IgG_1_-λ	0.25 mg
4	F	69	IgG_1_-λ	0.25 mg
5	F	59	IgG_1_-λ	0.25 mg
6	F	62	IgG_1_-κ	1.25 mg
7	F	50	IgG_1_-κ	1.25 mg
8	M	59	IgG_1_-κ	1.25 mg
9	M	57	IgG_1_-κ	1.25 mg
10	M	63	IgG_1_-λ	1.25 mg
11	M	52	IgG_1_-κ	2.5 mg
12	M	62	κ-light chain	2.5 mg
13	M	63	IgG_1_-κ	2.5 mg
14	F	60	IgG_1_-κ	2.5 mg
15	M	66	IgG_1_-κ	2.5 mg

### Clinical response of patients vaccinated with phage Id vaccine

**Figure 1:**
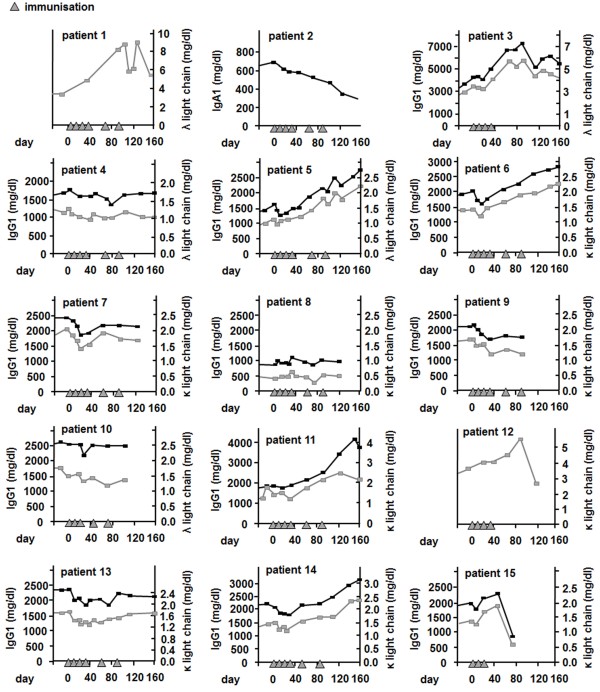
**Clinical response of patients vaccinated with Id-phage.** Paraprotein serum levels (black) and light chain excretion (gray) of each individual patient.

Clinical response was determined by serum M gradient and 24-hour light chain excretion measurement in patients identified as numbers 1–5, 6–10 and 11–15 receiving 0.25, 1.25 and 2.5 mg dosages of vaccine per treatment, respectively. The patients’ individual M gradient and 24-hour light chain excretion are displayed in Figure 1 and Table 2. A decrease or stabilization of paraprotein levels and/or 24-hour light chain excretion was apparent during treatment course in 11 out of 15 patients (patients 2, 3, 4, 5, 6, 7, 8, 9, 10, 13, 14). After vaccination with 0.25 mg phage Id vaccine, reductions of paraprotein levels were mainly observed during the first three weekly immunizations (patients 3–5), whereas in one patients suffering from IgA MM and treated with 0.25 mg phage vaccines containing light chains, the level of IgA paraprotein decreased 65% during treatment and follow up (patient 2). In contrast to the 0.25 mg-group, in which only two patients (patients 2 and 4) had stable paraprotein levels after three months, four of five patients having received the 1.25 mg vaccine dose displayed stable or reduced paraprotein concentrations in this time range (patients 7–10). In the 2.5 mg group, one patient showed reduction of paraprotein levels after treatment initiation and stable levels during follow up (patient 13), whereas another patient experienced reduction of paraprotein concentrations after the first three immunizations, followed by a re-rise of paraprotein levels thereafter (patient 14). The remaining patients in this high dose group (patients 11, 12 and 15) exhibited no clinical response to the phage Id vaccination.

**Table 2:** Paraprotein levels or 24-hour protein excretion in the urine before and after the vaccine treatment as well as the lowest level during treatment (mg/dl)

**No.**	**3 months before vaccination**	**Immediately before the first vaccination**	**Immediately after the last vaccination**	**Lowest level during treatment**	**3 months after the last vaccination**
1	1.57	3.43	8.19	3.43	-
2	0.51	0.64	0.36	0.36	0.30
3	1.57	3.43	4.01	3.30	5.75
4	1.17	1.26	1.11	0.99	1.23
5	0.54	1.07	1.86	1.03	2.27
6	1.10	1.46	1.91	1.20	2.45
7	1.61	1.99	1.78	1.45	2.12
8	0.55	0.43	0.58	0.34	0.57
9	1.51	1.66	1.46	1.20	1.46
10	1.68	1.79	1.42	1.22	1.66
11	0.89	1.35	2.43	1.20	1.88
12	2.84	3.54	3.99	3.54	5.40
13	1.90	1.65	1.67	1.29	1.81
14	1.17	1.46	1.81	1.17	2.34
15	1.07	1.40	1.90	1.33	-

**Figure 2:**
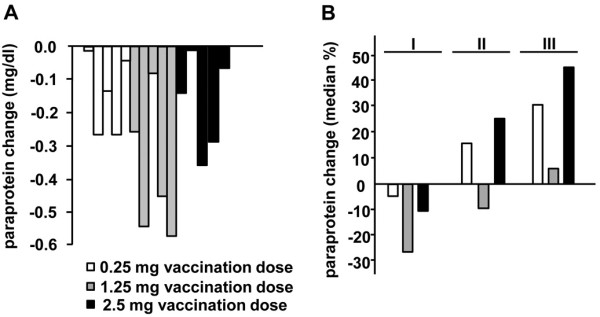
**Paraprotein changes during treatment and follow up. (A)** Maximum absolute paraprotein decrease of each patient. **(B)** Percental changes of paraproteins during treatment and follow up. (I) Maximum drop of paraprotein levels during treatment; median time after first vaccination: 88 days in the 0.25 mg group (range 6–148 days), 18 days in the 1.25 mg group (range 7–78 days) and 22 days in the 2.5 mg group (range 20–120 days). (II) Paraprotein levels immediately after the last vaccination in relation to paraprotein levels immediately before the first vaccination. (III) Relative paraprotein levels three months after the last vaccination in comparison to paraprotein levels immediately before treatment.

Figure 2 shows the best clinical response of all patients (A) and the relative changes of paraprotein levels during treatment and follow up in each treatment group (B). Patients in the 1.25 mg group displayed the best response with the greatest drop of paraprotein levels during treatment and the lowest paraprotein levels immediately after and three months after the last vaccination in relation to paraprotein levels detected before the first vaccination. However, these differences did not reach statistical significance in the limited number of study participants.

### Humoral immune response of patients vaccinated with phage Id vaccine

**Figure 3:**
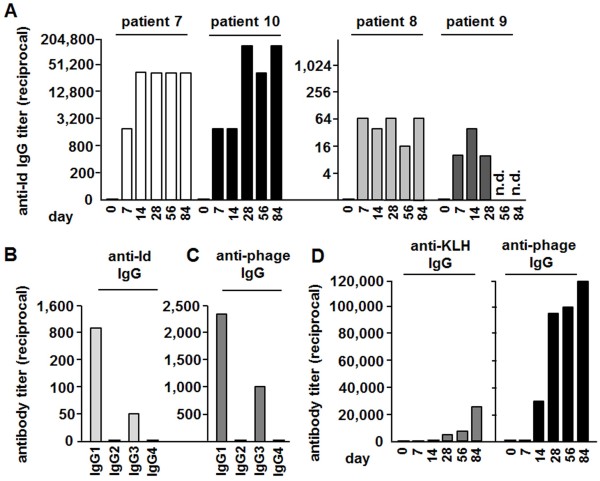
**Anti-Id, anti-phage and anti-KLH-specific IgG responses. (A)** Id-specific antibody response of patients 7–10 having received 1.25 mg phage Id vaccine. Patients’ sera were obtained shortly before each vaccination (day 1, 7, 14, 28, 56 and 84) and examined by ELISA using anti-Id antibodies. **(B)** Anti-Id specific immunoglobulin subtypes and anti-phage specific immunoglobulin subtypes **(C)** in patient 7 at day 28 after immunization with 1.25 mg phage Id vaccine. The results are representative for patients, who had raised and analyzed Id-specific antibodies (patients 7–10). **(D)** Antibody response to KLH (left) and wild type phage (right). Serum was taken shortly before each vaccination (day 1, 7, 14, week 4, 8, 12). Results of patient 2, having received 0.25 mg phage Id vaccination, are shown.

Anti-Id antibody levels and antibodies against the carrier components KLH and phage were determined in patients in the projected optimal 1.25 mg phage Id vaccine dosage group (patients 6–10) over the time course of administration (Figure 3). Patients exhibiting a clinical response to phage vaccines (patients 7–10) also raised tumor Id specific immunoglobulins. Antibody titers as high as 1:25,000 and 1:90,000 were found in patients 7 and 10, respectively. In this treatment group, 4 of 5 patients had a detectable anti-Id response. Patient 6 did not clinically respond to treatment and developed no measurable Id-specific humoral response (data not shown).

Both the Id-specific (Figure 3B) and phage-specific (Figure 3C) antibodies mainly comprised the IgG-isotype subtypes IgG1 and to a lesser amount IgG3, whereas IgG2 and IgG4 were only detectable in small amounts.

All treated patients demonstrated high levels of phage-specific antibodies (data not shown). The phage-specific antibody response was dose-dependent and detectable as early as 2 weeks after the first phage vaccination. In contrast, KLH-specific antibodies were only detectable in 80% of the patients. Furthermore, KLH-specific antibody levels were approximately ten times lower and only detectable two weeks later than phage-specific antibodies. Figure 3D shows the results of patient 2, having received 0.25 mg phage Id vaccination. The results are representative for study participants having received 0.25 and 1.25 mg phage Id vaccination. Regarding the immunoglobulin subtypes, a similar pattern with a predominant IgG1 response was observed in KLH-specific and phage-specific antibodies as compared to Id-specific antibodies (data not shown).

### Cellular immune response of patients vaccinated with phage Id vaccine

**Figure 4:**
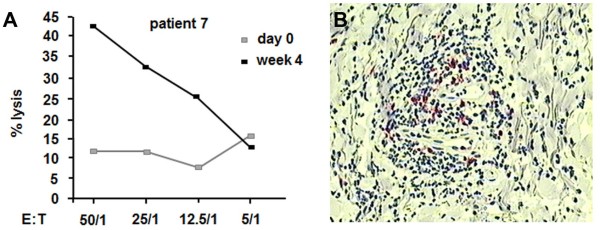
**Cellular anti-tumor response. (A)** Cytotoxic T lymphocyte (CTL) response in patient 7. Peripheral blood mononuclear cell cytotoxicity was determined by flow cytometry analysis against bone marrow cells comprising MM cells using an apoptosis detection kit. Target cells were taken from bone marrow biopsy samples from patient 7 before the first vaccination. PBMCs were collected 4 weeks after the first vaccination with 1.25 mg phage Id vaccine. E:T: effector/target cell ratio. **(B)** Skin biopsy oft the site of phage Id challenge of patient 7. CD8+ (red), but no CD4+ T cell infiltrate was demonstrated.

For measurement of cytotoxicity activity peripheral blood mononuclear cells (PBMCs) were collected at day 1 or 4 weeks after the first vaccination of the 1.25 mg phage vaccine dose from patient 7. These cells were used as effector cells for comparison against bone marrow biopsy samples containing MM target cells taken before the first vaccination. Use of the CyToxiLux apoptosis detection kit revealed that the PBMCs collected at 4 weeks were able to kill target myeloma cells across all effector to target cell ratios tested (Figure 4A), whereas PBMCs obtained at day 0 exhibited a low cytotoxic activity. At effector to target ratio 50:1, maximal lysis was 44% for the 4 week phage vaccine-primed PBMCs versus only 11% for PBMCs isolated at day 1, demonstrating the capacity of the phage idiotype vaccine to induce cytotoxic activity in a patient suffering from MM.

The patients’ cellular immune responses were further examined by delayed type hypersensitivity (DTH) tests against Id-phage, wildtype phage, myeloma Id protein and KLH (Table 3). DTH response was determined 48 hours after application of the respective antigens. All patients developed a strong DTH reaction against phage Id vaccine, wild type phage and KLH antigen, manifested by induration, redness and swelling at the site of challenge, whereas the reaction evoked against purified myeloma Id protein was weaker compared to the other antigens. However, in two patients having received the 1.25 mg page vaccine dosage (patients 7 and 10), DTH was unequivocally positive. Two more patients from the 1.25 mg-group displayed minor skin reactions indicating a positive vaccine response (patients 8 and 9). In order to characterize the type of infiltrating cells, a skin biopsy was obtained from the site of the challenge of patient 7. Immunohistochemical analysis revealed an infiltration of neutrophils, macrophages and lymphocytes, the latter representing CD8^+^ T cells (Figure 4B). In contrast, CD4^+^ T-cells and CD19^+^ B cells were not readily detectable as part of the DTH response.

**Table 3:** Results of the delayed-type hypersensitivity test; n.d. not done

**No.**	**Phage-idiotype vaccine**	**KLH**	**Wild type phage**	**Idiotype**
1	+++	+++	+++	-
2	+++	+++	+++	-
3	+++	+++	+++	-
4	+++	+++	+++	-
5	+++	+++	+++	-
6	+++	+++	+++	-
7	+++	+++	+++	+
8	+++	+++	+++	+
9	+++	+++	+++	+
10	+++	+++	+++	++
11	+++	+++	+++	-
12	+++	+++	+++	-
13	+++	+++	+++	+
14	+++	+++	+++	+
15	n.d.	n.d.	n.d.	n.d.

## Discussion

The present phase I/II study is the first trial employing a novel chemically linked phage-based Id vaccine targeting MM. We demonstrate a fast method for generating tumor-specific vaccinations with a maximal production success rate. Endotoxins were efficiently removed from phage preparations by an optimized purification protocol [7,9,10] and the resulting phage vaccine compositions could be readily employed in the vaccination studies. Previous methods based on generating a hybridoma cell line or PCR amplification of the Id from small numbers of tumor cells are costly and have failure rates as high as 15% with long production times of 3 to 13 months to vaccine completion [11,12].

We took advantage of the phage-inherent immunogenicity and the phage’s feature to allocate thousands of well-defined sites available for chemical conjugation [13]. The high molecular weight paraprotein was chemically coupled to the phage surface at high density. Preclinical exploration of the resulting phage vaccine revealed a superior immunogenicity compared to KLH-coupled Id and the genetically engineered phage expressing the Id on the major coat protein g8 [7]. In the present study, the humoral response against phage was raised earlier and more efficiently than against the well-known KLH, which was used as control antigen. Thus, although KLH-coupled Id still represents the gold standard for Id vaccinations and has been shown to provide protection in the murine tumor challenge model [14,15], chemically conjugated Id-phage may have the potential of a superior immunogenicity.

So far, clinical trials employing active anti-Id vaccinations were largely disappointing with mixed responses [16]. Several phase I/II studies documented clinical response [11,17-22] with e.g. complete elimination of tumor cells carrying the t(14;18) translocation in 8 out of 11 patients with minimal residual follicular lymphoma [3]. In other studies, Id-KLH vaccines failed to elicit an anti-Id immune response in more than half of the patients [4,12,21]. In Id vaccine trials with myeloma patients using Id-pulsed dendritic cells amongst others, altogether about 55% of patients mounted an Id-specific response and about 12% displayed a clinical response [23,24]. Clinical response was difficult to evaluate in the majority of studies, because vaccines were mostly conducted in a state of minimal residual disease based on the presumption derived from animal studies that vaccinations are more successful in a state of low tumor burden, entailing that most patients had previously received cytoreductive and immunosuppressive therapy [16]. The present study was designed as clinical phase I/II trial primarily serving to determine safety, toxicity and initial response. As a consequence, patients allowed to participate by the approval of the local ethics committee were patients with advanced disease suffering from a relapse or partial remission after high dose chemotherapy. Due to a high tumor load and subsequently impaired immune function in myeloma patients, for whom the bone marrow as the predominant site of tumor growth progressively loses its ability to procure a normal pool of immunological active cells, these patients were not expected to respond to active immunotherapy. Moreover, preclinical studies suggest that high circulating paraprotein amounts are associated with unresponsiveness to Id vaccination [25]. Nevertheless, treatment with the phage Id vaccine led to a clinical response in several patients, reflected by a reduction or stabilization of paraprotein levels. These effects were associated with the induction of anti-Id antibodies in 4 of 5 patients after vaccination with 1.25 mg phage vaccine, whereas a further dose escalation to 2.5 mg did not increase efficacy. The 2.5 mg dose group displayed a weaker response than the 1.25 mg dose group, which we rate as incidental finding due to the low patient number. Remarkably, Id-specific antibodies were developed in several patients despite the presence of high paraprotein levels and despite the assumption that most of the mounted anti-Id antibodies exist in patients’ sera in the form of immune complexes bound to the serum paraprotein, emphasizing the superior immunogenicity of the present phage vaccine formulation.

Besides the induction of Id-specific antibodies, cellular immune response is of importance for an effective anti-tumor response. In treatment-naive patients with indolent B cell lymphoma, only cellular-mediated responses correlated with superior progression-free survival and durable objective remissions [26]. In MM patients, the cellular immune response is especially crucial, since myeloma cells secrete their tumor-specific immunoglobulins and thus the anti-Id humoral immune response may result in binding and neutralizing of anti-Id specific antibodies by soluble paraproteins [27]. Accordingly, it was found previously that reduction of circulating myeloma cells correlated with vaccine-induced Id-specific T cell responses [28]. Thus, it is fundamental that phages are able to be incorporated by antigen-presenting cells and to induce not only a humoral, but also a cellular immune response [29,30]. Uptake and processing of the phage-coupled Id protein and subsequent presentation via both the MHC I and II is likely. Antibody subtype analysis in the phage-vaccinated patients revealed that detected anti-Id antibodies comprised mainly the subtype IgG1 and to a lesser extend IgG3, suggesting that a Th1-like T cell response is elicited by phage Id vaccination, since it is known that IgG1 and IgG3 isotypes promote antibody-dependent cellular cytotoxicity and complement-dependent cytotoxicity [31]. This is consistent with the fact that interferon-γ, a cytokine produced by Th1 cells, enhances IgG1 production in humans [32]. The positive anti-Id specific skin reaction of patients displaying an anti-Id specific humoral response confirms the induction of a cellular immune response. Immunohistochemical analysis of the DTH lesions revealed an infiltration of neutrophils, macrophages and lymphocytes, which were mainly CD8^+^ T cells, indicating that Id-conjugated phage vaccines generate an effective cytotoxic T cell response.

It is still impossible to determine in advance which patients will respond to the costly Id vaccines. Naturally, response to treatment depends on the immune response generated towards the Id [33] and thus it is not surprising that only patients developing antibodies against their own Id have shown benefit with regard to time to progression and overall survival in previous clinical studies [16,33,34]. Accordingly, we demonstrate a correlation of clinical response and anti-Id humoral immune response. Additionally, the phage vaccine dose seemed to play a role for induction of Id-specific antibodies. Furthermore, another study including patients with follicular lymphoma found that the vaccine isotype of the fragment crystallizable region may affect immunogenicity, as IgM-Id-KHL improved disease-free survival, whereas IgG-Id-KLH vaccination had no effect [6]. An explanation for the latter finding may be that the fragment crystallizable region of IgG has highly promiscuous MHC class II T cell epitopes specifically activating regulatory T cells and thus skewing immune response towards tolerance rather than immunogenicity [35]. However, neither performance scores nor response to chemotherapy or percentage of T cell subsets in tumor biopsy specimens were valid markers to distinguish patients with or without anti-Id response. It is also unlikely that the Id-specific immune response is only dependent on differences in immune competency, because most patients mount a substantial immune response to KLH [34], which was confirmed in the present study.

While phage-specific antibodies were detectable in all patients of the present study, only a subset of patients developed anti-Id antibodies. Hence, Id immunogenicity is still insufficient within this cohort of patients, which was also reflected by a weaker skin reaction evoked by purified myeloma Id protein in comparison to other antigens. Further strategies to improve the Id immunogenicity are under investigation, but still have to prove efficiency in the clinical setting. Phage may be further optimized by design, for example by co-expression of fragments or antigenic determinants promoting the uptake of bacteriophages by dendritic cells [36]. On the other hand, genetic removal or modification of immunodominant regions of coat proteins was demonstrated to focus and improve the epitope-specific immune response by decreasing the antigen complexity of the phage surface [37]. From a pathophysiological point of view, strategies to overcome T cell tolerance by checkpoint blockade inhibitors may be well suited for combination with vaccinations. Cytotoxic T-lymphocyte antigen 4 (CTLA4) ligation is important for the immunosuppressive function of regulatory T cells and anti-CTLA4 antibodies produced encouraging results in melanoma patients [38]. An alternative target is the PD-1/PD-L1 axis. PD-1 (programmed cell death protein 1) is expressed by activated T cells and binds to its ligand PD-L1 that is expressed on potential target cells, thereby rendering the T cell unresponsive [39-41]. Expression of PD-1 and its ligands in the microenvironment of myeloma and recent data indicating a role of the PD-1 pathway in the immune evasion by myeloma cells make therapeutic PD-1/PD-L inhibition an interesting option in multiple myeloma [42]. However, severe toxicities were reported after use of checkpoint blockade inhibitors, including 23% grade 3 or 4 adverse effects, such as inflammatory colitis and hypophysitis after use of anti-CTLA4 antibodies and cardiomyopathy and lung infiltrations after application of anti-PD-L1/PD-1 antibodies [38,43].

## Conclusion

While refinements for increasing the immunogenicity of the target Id may still be needed, we conclude from our data that myeloma Id paraproteins chemically conjugated to phage particles appear to be suitable for use as vaccines and capable of evoking tumor-specific immune responses. The current study demonstrates the feasibility to rapidly create tumor-specific phage vaccines for each individual patient. The present data may be helpful and encouraging for the design of larger clinical trials involving MM patients, preferably earlier in their disease course, e.g. during first remission, when the immune function is not yet severely impaired by extensive clonal plasma cell proliferation and multiple chemotherapies and thus a better response to active vaccination can be expected.

## Abbreviations

CTLA4: Cytotoxic T-lymphocyte antigen 4; DTH: Delayed type hypersensitivity; ELISA: Enzyme-linked immunosorbent assay; g3: Minor coat protein g3; g8: Major coat protein g8; GM-CSF: Granulocyte-monocyte colony stimulating factor; Id: Idiotype; Ig: Immunoglobulin; KLH: Keyhole limpet hemocynanin; M: Mol/liter; MHC: Major histocompatibility complex; MM: Multiple myeloma; NaCl: Sodium chloride; n. d: Not done; PBS: Phosphate-buffered saline; rpm: Round per minute; PBMCs: Peripheral blood mononuclear cells; PD-1: Programmed cell death protein 1; s.c: Subcutaneously.

## Competing interests

The authors declare that they have no competing interests.

## Authors’ contributions

TR conceived of the study, planned its design and coordination and analyzed the data. CT analyzed the data and wrote the manuscript. WN participated in the design of the study, analyzed the data and helped to draft the manuscript. CB performed parts of the experiments. TB analyzed the data and helped to draft the manuscript. MD and TB participated in the data collection and analysis. MF analyzed the data. CB performed parts of the experiments and analyzed the data. FO planned the study design, managed and coordinated the process of data analysis and drafting of the manuscript. All authors read and approved the final manuscript.

## Supplementary Material

Additional file 1 Adverse events observed in patients after phage Id vaccination. Click here for file
