# Supplementary material for: Phage idiotype vaccination: first phase I/II clinical trial in patients with multiple myeloma
Source: J Transl Med. 2014 May 9;12:119. doi: 10.1186/1479-5876-12-119 (PMC4113220; doi:10.1186/1479-5876-12-119)
Supplement: Additional file 1 — Adverse events observed in patients after phage Id vaccination. [file 1479-5876-12-119-S1.docx]

**Additional File 1** Adverse events observed in patients after phage Id vaccination.

| Parameter | CTC Grade 0 | CTC Grade 1 | CTC Grade 2 | CTC Grade 3 | CTC Grade 4 |
| --- | --- | --- | --- | --- | --- |
| Allergic reaction | 6 | 8 | 1 | 0 | 0 |
| Allergic rhinitis | 10 | 0 | 0 | 0 | 0 |
| Autoimmune reaction | 10 | 0 | 0 | 0 | 0 |
| Allergy/Immunology-Other | 10 | 0 | 0 | 0 | 0 |
| Hemoglobin | 7 | 1 | 1 | 1 | 0 |
| Hemolysis | 15 | 0 | 0 | 0 | 0 |
| Sinus tachycardia | 13 | 2 | 0 | 0 | 0 |
| Hypertension | 13 | 2 | 0 | 0 | 0 |
| Hypotension | 12 | 2 | 1 | 0 | 0 |
| Fatigue | 11 | 4 | 0 | 0 | 0 |
| Fever | 8 | 2 | 0 | 0 | 0 |
| Rigors, chills | 8 | 2 | 0 | 0 | 0 |
| Flushing | 2 | 0 | 0 | 0 | 0 |
| Injection site reaction | 0 | 0 | 10 | 0 | 0 |
| Pigmentation changes | 9 | 3 | 0 | 0 | 0 |
| Wound- infectious | 0 | 1 | 0 | 0 | 0 |
| Impaired Vision | 0 | 0 | 0 | 1 | 0 |
| Diarrhea | 7 | 3 | 0 | 0 | 0 |
| Metabolic/Laboratory | 10 | 0 | 0 | 0 | 0 |
| Chest pain | 8 | 4 | 0 | 0 | 0 |
| Arthritis/Arthalgia | 7 | 3 | 0 | 0 | 0 |
| Headache | 8 | 4 | 0 | 0 | 0 |
| Myalgia | 7 | 3 | 0 | 0 | 0 |
| Creatinine | 0 | 0 | 0 | 1 | 0 |
